# Proximity Sensor for Measuring Social Interaction in a School Environment

**DOI:** 10.3390/s24154822

**Published:** 2024-07-25

**Authors:** Tania Karina Hernández-Heredia, Cesar Fabián Reyes-Manzano, Diego Alonso Flores-Hernández, Gabriel Ramos-Fernández, Lev Guzmán-Vargas

**Affiliations:** 1 Unidad Profesional Interdisciplinaria en Ingeniería y Tecnologías Avanzadas, Instituto Politécnico Nacional, Mexico City 07340, Mexico; tkhernandezh@ipn.mx (T.K.H.-H.); dfloreshe@ipn.mx (D.A.F.-H.); 2 Tecnológico Nacional de México, Tecnológico de Estudios Superiores de Ixtapaluca, Km. 7 Carretera Ixtapaluca-Coatepec S/N San Juan, Ixtapaluca 56580, Mexico; cesar.rm@ixtapaluca.tecnm.mx; 3 Instituto de Investigaciones en Matemáticas Aplicadas y en Sistemas, Universidad Nacional Autonoma de Mexico, Mexico City 04510, Mexico; gabriel@aries.iimas.unam.mx; 4 Centro de Ciencias de la Complejidad, Universidad Nacional Autonoma de Mexico, Mexico City 04510, Mexico

**Keywords:** interaction, sensors, spatial configuration, received signal strength indicator

## Abstract

Social interactions are characterized by being very diverse and changing over time. Understanding this diversity and dynamics, as well as their emerging patterns, is of great interest from social, health, and educational perspectives. The development of new devices has been made possible in recent years by advances in applied technology. This paper presents the design and development of a novel device composed of several sensors. Specifically, we propose a proximity sensor integrated by three devices: a Bluetooth sensor, a global positioning system (GPS) unit and an accelerometer. By means of this sensor it is possible to detect the presence of neighboring sensors in various configurations and operating conditions. Profiles based on the Received Signal Strength Indicator (RSSI) exhibit behavior consistent with that reported by empirical relationships. The present sensor is functional in detecting the proximity of other sensors and is thus useful for the identification of interactions between people in relevant contexts such as schools.

## 1. Introduction

Proximity sensors are devices designed to detect the presence or absence of an object within a certain range without physical contact. In general, the operation of this type of sensor is based on the principle of detecting changes in electromagnetic fields [1,2,3], i.e., changes in the signals emitted and/or received to determine the proximity of an object. These types of sensors have been used in various fields such as automotive, electronics, public safety and robotics, among others [4,5,6,7]. To date, there are several types of proximity sensors ranging from photoelectric to magnetic sensors. In the context of the study of social interactions, the use of sensors to detect interactions has been very useful in several studies [8,9,10,11]. In [12], a wearable sensor based on semi-supervised learning is presented to identify the interaction between university students to analyze the risk of exposure to SARS-CoV-2 transmission. The sensor is attached to the participant through a headband, including an image detector, an IR projector, and an RGB sensor. In addition, the components such as a power source and a microcomputer are mounted in the pocket of a specific jacket, reducing the portability and affecting the participant’s feeling in the tests. Or, they are used to record data to perform various analyses in future studies, as shown in [13]. These sensors provide valuable data on social contact patterns and interactions within communities. Studies have shown that proximity sensors can capture high-resolution temporal data, allowing for detailed analysis of social dynamics and contact patterns [8]. For instance, radio frequency identification (RFID), which uses radio waves to transmit data wirelessly between an RFID transponder and an RFID reader, has been used to identify face-to-face interactions in many recent studies [10,14,15,16]. For example, Génois et al. [17] present the data collected at four science conferences for social interaction studies where the participants use cards and antennas with RFID technology, detecting other sensors in neighborhoods of a 1.5 m radius in face-to-face contact. This tool has the limitation of needing to be in certain areas with no obstacles in its way to be detected in order to obtain accurate measurements. One of the options that has often been used to identify proximity is Bluetooth, especially in cases where it is necessary to identify social interactions in controlled conditions. On the other hand, in [18], a face-to-face interaction study is presented using an IoT-based framework employing Bluetooth communication to represent social closeness and analyze the dynamics of social relationships through various models. Bluetooth proximity sensors provide some advantages such as low power consumption, cost-effectiveness and compatibility with a wide range of devices [19,20,21,22,23]. However, the accuracy of proximity detection can vary depending on factors such as signal strength, interference and the environment in which the sensors are deployed. The use of Bluetooth to identify social interactions has certain limitations because proximity can be misinterpreted as an interaction. However, this information can be extended by adding other sensors that can capture additional information such as angles of orientation, tilt and acceleration, so that if a representative displacement or rotation occurs, this information is useful for the detection of an interaction.

Since there exist limited options to obtain commercially available, low-cost proximity sensors that are adequate to detect social interactions, we present a viable option that fulfills these criteria [24,25,26,27,28]. Of particular interest is identifying social interactions in settings such as schools, where interactions may eventually impact the learning process and school performance. We propose a proximity sensor that consists of the integration of three devices: a Bluetooth sensor, a global positioning system (GPS) unit and an accelerometer. The components chosen in this work are from different companies and of commercial use.

In our tests, using the information obtained by the integrated devices, we have been able to detect the presence or proximity of neighboring sensors in various configurations and operating conditions. Specifically, we were able to reproduce the typical behavior of profiles based on the Received Signal Strength Indicator (RSSI), which is an indicator of the power level received by an antenna or receiver, for certain wireless sensor networks. We tested the sensor’s performance by means of two experiments. The first one consisted in registering the RSSI strength between two sensors, increasing the distance among them, while the second involved measurements from eight sensors working simultaneously, placed in a specific star-type topology. From our experiments, which are based on the established configuration (master-slave) on each sensor, we were able to obtain the following results:

- High reading quality between the sensors.
- Minimum amount of loss of data read.
- Fast detection between sensors.
- Stability in the sensing range.

The remaining structure of this article is as follows. In Section 2 we present the main characteristics of the elements that constitute the sensor, as well as a brief explanation of their operation. In Section 3, we describe the basic aspects of the sensor operation, followed by the results (Section 4), where we describe the two experiments performed. Finally, we include our conclusions in Section 5.

## 2. Materials and Methods

**Figure 1:**
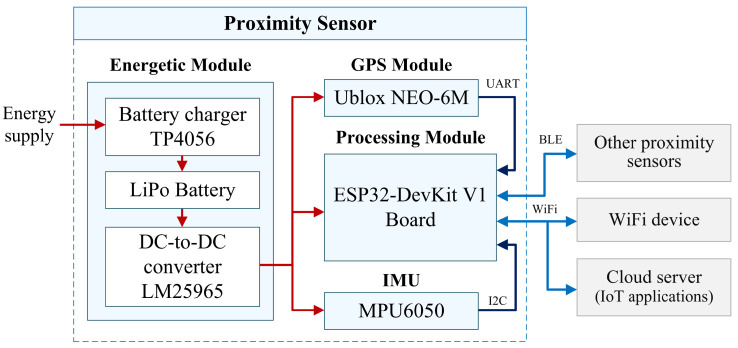
Physical architecture of the proximity sensor, including electrical power connections (red arrows) and data transfer (blue arrows).

The development of the proximity sensor was based on the methodology proposed in [29], which uses a systemic and concurrent approach, allowing performance improvement through constant validation and verification processes. Based on the definition of the problem, we built the functional architecture of the sensor, specifying the functions that the sensor should perform. Subsequently, we developed the physical architecture (see Figure 1), which is a description of the resources that the system comprises, grouped by modules. The sensor is made up of four modules:

1. Processing module: It is composed of an ESP32-DevKit V1 board, which performs the functions of information processing, decision making, signal conditioning, and internal and external connectivity of the proximity sensor. The ESP32 microcontroller of the board integrates Bluetooth, Bluetooth LE, and Wi-Fi, ensuring a wide range of applications with greater adaptability. The Wi-Fi channel allows a direct connection to the internet, while the Bluetooth module allows connecting and transmitting low-energy beacons for detection. The quiescent current is less than 5 μA, which makes it suitable for portable electronic applications as it is battery-powered. The module supports a data rate of up to 150 Mbps and an adjustable transmission power of 20 dBm at the antenna. The combination of features of the dual-core processor, Wifi and Bluetooth connectivity, as well as its general purpose pins (GPIO), provide the possibility of developing various applications, including IoT applications. The ESP32 can be programmed in several languages. In our case, we used the C language within the Arduino Integrated Development Environment (IDE), which provides a large number of libraries and tools for using the microcontroller in various contexts. The programming of the sensor includes particular functions that were programmed using different libraries allowed by the Arduino environment, so the sensor can work autonomously as long as it has the minimum energy needed to power all the devices that compose it. It is important to recall that the power supply of each of the sensors is different and therefore requires a specific current (≈850 mW) to be able to work and function optimally. The tests performed with the sensor showed that it is necessary to maintain the mentioned current in the battery as a minimum. In case this requirement is not met, the sensor cannot complete the requested functions and stops communicating even though it may physically show that it is on.
2. Energetic module: This module performs the functions of storing electrical energy, conditioning, and distributing the energy to the other components of the proximity sensor. It is made up of the following components: a 3.7 V Lithium-Polymer (LiPo) battery with a capacity of 1200 mAh [30], a Standalone Linear Li-ion Battery Charger that is powered by a mini-USB port at 5 V [31], and a DC-DC switching boost converter that assists in controlling the battery output to a voltage of 3.3 V [32].
3. GPS module: This module determines the real time position of the sensor and monitors the movement, providing coordinates of the sensor in open spaces or near windows. This information can be useful to reconstruct the path of the sensor, which can help in the identification of a social interaction and accurate time measurements. The GPS module based on the Ublox NEO-6M chip, includes a GPS antenna with UFL connector, a small battery and an EEPROM memory that allows saving of the last positioning data [33]. The supply voltage is 5 V, it has a search consumption of 67 mA and a tolerance of ±2.5 m [34]. This sensor measures longitude and latitude with a sampling rate of 0.2 Hz and sends the data through a UART port configured at a rate baud of 9600 bps.
4. Inertial Measurement Unit: It has the function of integrating motion information based on acceleration and angular velocity data acquired from the accelerometer and gyroscope, respectively. This enables calculation of inertial navigation as well as vibration, drop detector, distance and velocity measurement. The MPU6050 sensor is an IMU with 6 Degrees of Freedom (DoF) and is composed of a 3-axis accelerometer and a 3-axis gyroscope, which measure acceleration [m/s^2^] and angular velocity [deg/s] in the *x*, *y*, and *z* axes, respectively. This sensor is powered by a 5 V power supply and was configured for a measurement range of 8 g in the accelerometer and 500 deg/s in the gyroscope. It also has a digital motion processor (DMP) that allows the calculation of the position of the sensor in pitch angle, roll angle, and yaw angle [35].

**Figure 2:**
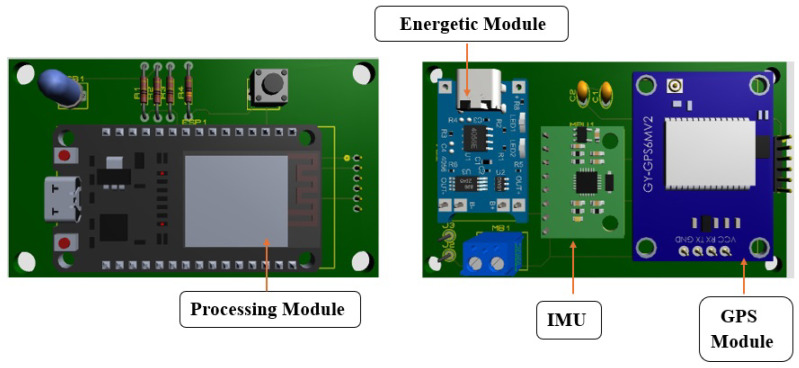
Design of the electronic hardware for the modules integration, top view (**left**) and bottom view (**right**).

Finally, once we analyzed the sensors that will integrate our proximity sensor, we carried out a unification process, which ensured that the operation of each module operated in an integrated manner. This is relevant because each of the modules operate with particular characteristics designed by the manufacturer, so that when unifying them, it must be ensured that they work within the range of the specifications indicated by the manufacturer. Figure 2 shows the design of the proximity sensor and the integrated modules.

## 3. Proximity Sensor Operation

### 3.1. Sensor Behavior

There are different electronic modules (sensors) on the market that can be integrated for the purposes of our proximity sensor. Therefore, when developing the present sensor, we have considered not only addressing a current need, but also ensuring that it can eventually be used in different conditions. Having a complete sensor that satisfies most of the needs is a complex task, so in our case we have considered the integration of several modules that are efficient in acquiring the key information for the identification of a social interaction.

The grouping of these electronic modules results in our proximity sensor based on parameters such as location, acceleration-gyroscope, and communication; as well as the possibility of bilateral BLE communication and RSSI measurement with other sensors. This information is achieved through Arduino programming and the use of libraries, where the ESP32 performs a scan every 5 s to detect nearby devices with its BLE module in Beacon configuration. This identification is conducted by assigning names in the format set in the programming (e.g., E[0−9][0−9][0−9]). Thus, once the contact is established, the receiving sensor retrieves the name of the identified device, the RSSI of the signal and its MAC address. In addition, it obtains the information registered by the GPS and the IMU, identifying the presence of the other sensors in its vicinity.

The sensor communicates through Bluetooth Low Energy (BLE) and Universal Asynchronous Receiver/Transmitter (UART), which allow asynchronous transmission and reception of data. This enables the connection to the Global Positioning System, allowing it to read signals transmitted by the satellite. On the other hand, the I2C Inter-Integrated Circuit connects to the IMU, which provides motion information (acceleration and angular velocity). All these modules are powered by an energy module containing a Lipo battery connected to a converter and its power module.

**Figure 3:**
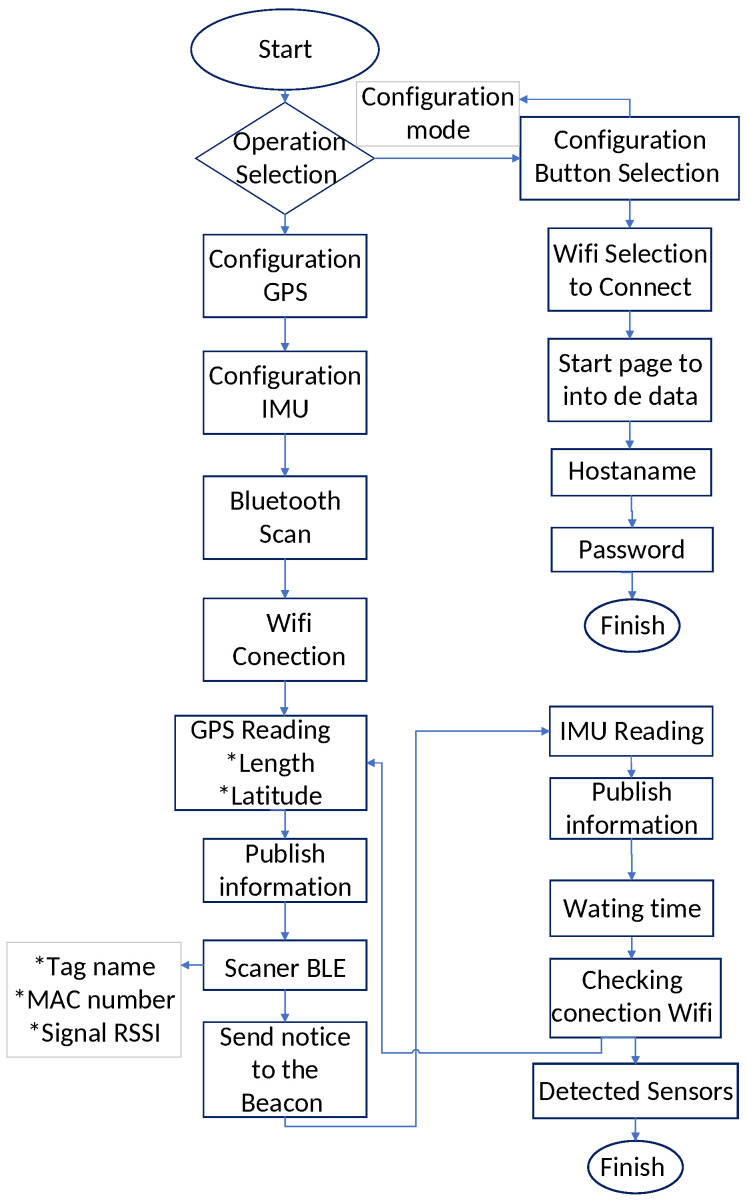
Flow diagram showing the configuration and working modes of the sensor.

Figure 3 shows the flow diagram of the operation of the proximity sensor. In this diagram we can see the operating mode of the sensor and its configuration, which is necessary for an efficient detection of nearby devices. In order to explain the flow diagram in a general way, we begin by mentioning that the programming of the sensor is in series, that is, the programming is indicated in such a way that it must comply with the indications line by line. The way to proceed is as follows: When starting the sensor functions you have the possibility of accessing two modes: (i) Configuration mode or (ii) Data detection. To enter mode (i), it is necessary to detect a physical input (push button), giving access to the sensor and providing the necessary configuration parameters for the sensors. The parameters used are as follows: Name of the network to be connected; Name of the sensor and Port number.

Regarding mode (ii), this consists of configuring the sensors that make up the proximity sensor, that is, configuring the GPS, IMU, and Bluetooth of the ESP32. At the end of this process, the sensor connects to the configured network to send the readings of its next activity. Immediately after, it starts reading and publishing the GPS data (latitude and longitude). It also scans the Bluetooth BLE (name, MAC address, and RSSI(dB)). After this action, the IMU starts to operate and the readings obtained by the IMU are published. To obtain a separation between the readings, a timeout of 2 s is assigned. Then, the Wi-Fi connection is checked again and the cycle is repeated.

As can be seen in Figure 3, the process consists of a serial cycle, which limits the execution of any indication due to interruptions or possible interferences. For this reason, an indicator LED (RGB) was placed at specific points in the configuration, allowing the user to know the current state of the system. In addition, this configuration allows for obtaining power measurements (RSSI), indicating the power that can be registered by the sensors of the receiving system.

### 3.2. Sensor Calibration and Error Reduction

Due to the limited resolution of RSSI measurements, granularity errors can arise, which refer to inaccuracy or lack of precision in locating a device [36]. This type of error manifests mainly at distances greater than 0.5 m, where measurements tend to blend. To reduce these errors, an averaging filter is applied before calculating the distance to minimize fluctuations and obtain better resolution. In addition, each distance calculation is performed using two sets of measurements, one for each sensor. Another challenge is the multipath effect, where signals collide with obstacles present in the environment and are reflected, merging with the original signal, known as non-line-of-sight (NLOS) [37]. Since the sensors have been designed for people in motion, mapping the environment, as proposed by some authors, is not very functional [38,39]. One solution to overcome this problem is to rely on inertial sensors to obtain an approximation of the person’s direction (based on a natural forward walking motion) and to give more weight to the sensors located just in front. On the other hand, packets are filtered based on the sensor name to avoid analyzing packets sent by the same sensor. Other relevant parameters to consider are weather conditions such as temperature and relative humidity, which influence RSSI measurements [40]. It has been shown that temperature has a negative correlation with RSSI at temperatures above 0 °C, with a loss of 1.3 to 2 dBm for every 10 °C increase [41,42,43]. In contrast, relative humidity (RH) has a positive correlation with RSSI, increasing approximately 0.3 dBm for every 10 °C increase in RH. It has also been shown that at temperatures below 0 °C, these values can be three times higher [42,44].

Finally, to avoid device heterogeneity errors, which occur when there are differences in device hardware, we use modules and components with the highest compatibility based on the manufacturer’s specifications. Additionally, each sensor is calibrated to minimize errors due to device heterogeneity and environmental conditions. The calibration consists of using two sensors (S1 and S2) placed on a table in a classroom (an environment similar to where the future experiments will be performed). S1 is placed at the starting point, and S2 is placed at 1 m with respect to S1. RSSI measurements are registered in the position for 8 minutes. In this way, the reference value is obtained and later used to calibrate the other sensors (see details of the experiment (i) in Section 4).

## 4. Experimental Results

### 4.1. Experimental Setup

The RSSI behavior can be modelled by the so-called Friis transmission equation [45,46], which establishes the behaviour of the power of the signal recorded by a receiver as a function of distance. Formally, the Friis equation is given by $RSSI\left(D\right)=P_{r1}-\kappa \log_{10}\left(D\right)$, where $P_{r1}$ is the received power at one meter, κ is a “path loss” parameter related with the transmission media and *D* is the distance. To empirically characterize the behavior of signal strength as a function of distance, we have performed two controlled experiments: (i) when a pair of sensors are placed on the ground and controlling the distance between them; and (ii) when a group of eight sensors are placed on the ground in a predetermined network-like configuration.

The physical and time conditions of the scenario where the experiments were carried out can be seen in Table 1:

### 4.2. Experiment (i)

**Figure 4:**
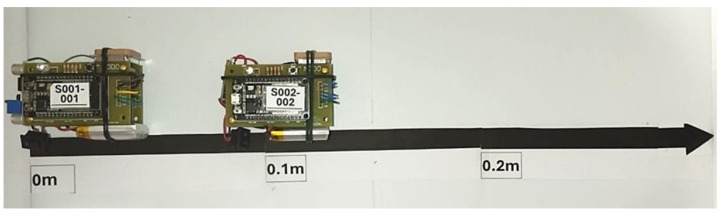
Image of the physical location of the sensors S1 and S2 during the measurement of the mutual signals for two distance values.

**Figure 5:**
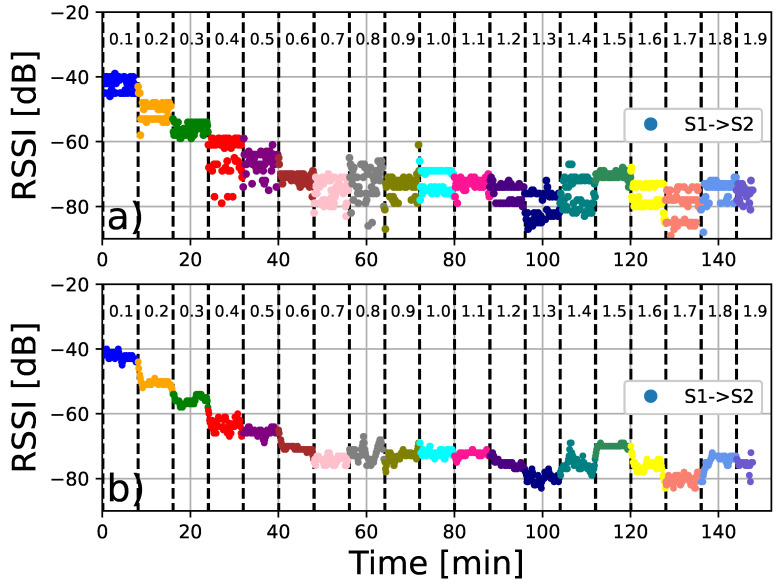
RSSI signal strength for several distance (meters) values and time intervals. (**a**) Scans of sensor 1 using the data emitted by sensor 2. The values shown are measurements per distance performed every five seconds during eighth-minute time intervals. (**b**) Similar to (**a**), but showing the mean values obtained from a moving average with one-minute windows.

**Figure 6:**
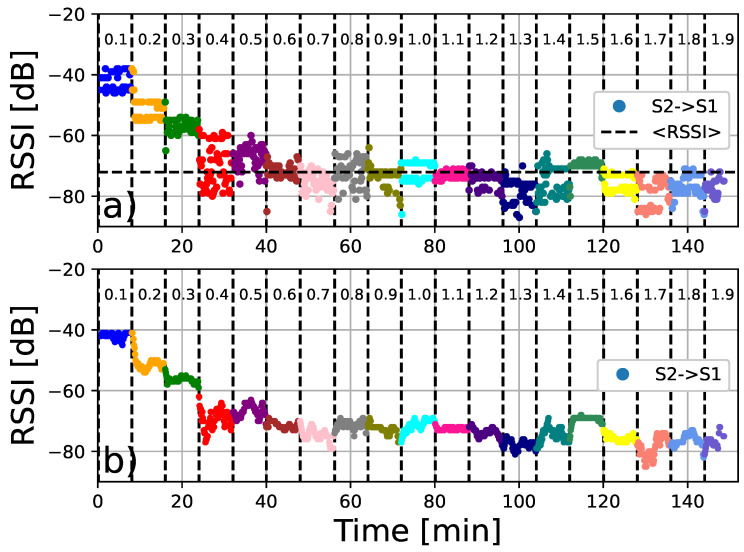
RSSI signal strength for several distance (meters) values and time intervals. (**a**) Scans of sensor 2 using the data emitted by sensor 1. The values shown are measurements per distance performed every five seconds during eight-minute time intervals. The reference value ${\langle RSSI\rangle }_{1m}\approx -68$ is also shown, which corresponds to the average value at a distance of 1 m. (**b**) Similar to (**a**), but showing the mean values obtained from a moving average with one-minute windows.

Two devices are placed on the ground (Sensor 1 and Sensor 2), where we can control the relative distance between them, as shown in Figure 4. Our strategy is based on recording the interactions between the sensors during a certain time interval (8 mins.). Initially, they were placed 10 cm apart and this distance is increased by 10 cm, up to a maximum distance of 2 m. The resulting measurements are presented in Figure 5 and Figure 6. In particular, Figure 5 shows the signal strength of the signal detected by sensor 1 coming from sensor 2 while the opposite is plotted in Figure 6. We observe that the measured signal intensity fluctuates with a representative mean value. For small distance values (D≈ 10 cm), the signal strength is relatively high −40 dBm, and with increasing distance, the signal strength decreases to values close to −80 dBm. We observe that the RSSI response provided values ranging from −40 to −90 dBm. According to RSSI quality tables [47], values greater than −65 dBm represent high quality, −65 dBm to −75 dBm represent good quality, −75 dBm to −85 dBm represent the limit of useful quality, and lower values represent poor data. Another important parameter for calibration purposes is the RSSI value when the separation between the sensors is 1 m. As shown in Figure 6, the average value ${\langle RSSI\rangle }_{1m}=-68.07\pm 2.27$ represents the reference value for the calibration of the other sensors.

**Figure 7:**
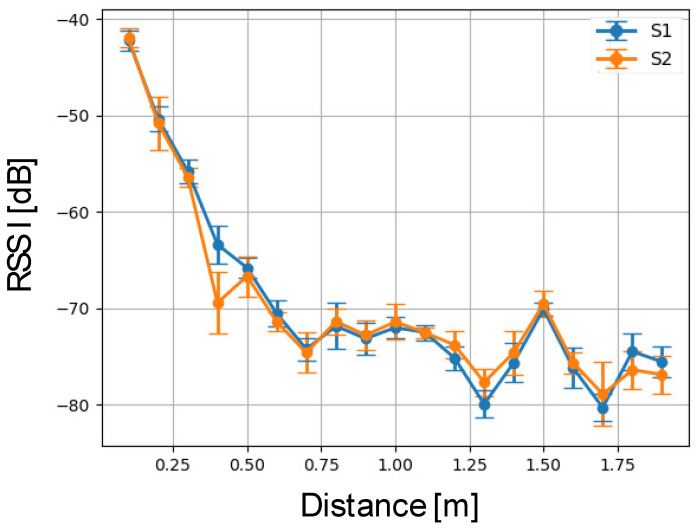
Signal strength vs. distance for the measurements from sensors S1 and S2. We observe that the signal intensity decreases as the distance increases. The vertical bars represent the standard deviation over the set of measurements.

**Figure 8:**
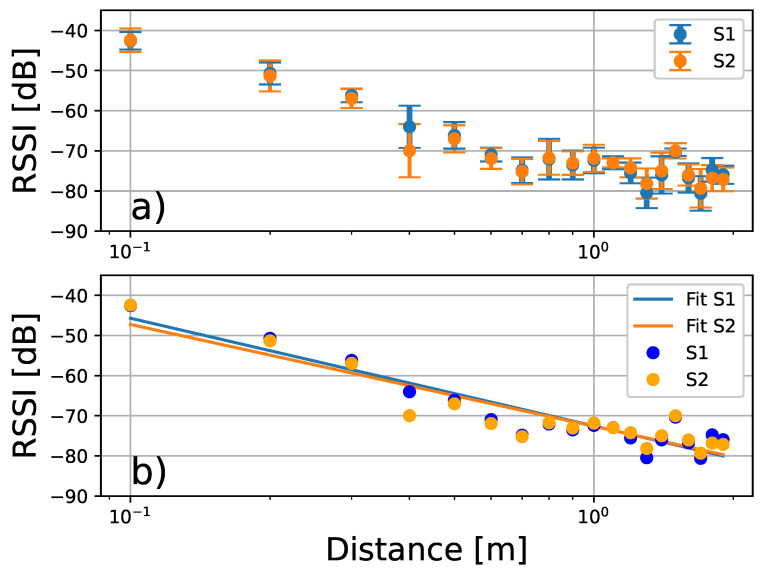
(**a**) Logarithmic-linear representation of the signal strength (RSSI) vs. distance for data obtained from sensors S1 and S2. This plot shows that the decay is logarithmic with an approximate linear behavior in this semi-log plane. (**b**) The calculations of the linear regression yield the values for the slopes: $a_{1}=-1.16$ ($R^{2}=0.879$) for S1 and $a_{2}=-1.10$ ($R^{2}=0.851$) for S2.

For a better identification of the signal intensity decay, we have calculated the mean value and standard deviation of each interval shown in Figure 7. It can be seen that both measurements present a similar profile as the distance increases. Then, using the mean values of the signal intensity and considering the different values of the distance, we performed a linear regression to identify the constants that characterise the straight line in a semi-logarithmic plane (RSSI=b+alog(D)). These results are shown in Figure 8. The calculations lead to the following values for the parameters: $a_{1}=-1.16$ and $b_{1}=-7.25$ ($R^{2}=0.879$) for the data detected by S1, while the data sensed by S2 result in $a_{2}=-1.10$ and $b_{2}=-7.26$ ($R^{2}=0.851$); see Figure 8b. These responses agree with the values predicted by the Friis equation [46]. In particular, $a_{1}=-1.16$ and $a_{2}=-1.10$ correspond approximately to the values of the path loss parameter given in refs. [46,47,48].

It is important to mention that the behavior of the signal intensity measured by the device allowed us to determine an effective threshold value (in dBm) to identify sensors that, based on the received values, can be distinguishable and represent a social interaction. In our case, we choose RSSI = −75 dBm as the threshold value, which is approximately 1.5 m. The interval 0–1.5 m is in general agreement with previous studies suggesting that most social interactions occur within this interval [49,50].

### 4.3. Experiment (ii)

**Figure 9:**
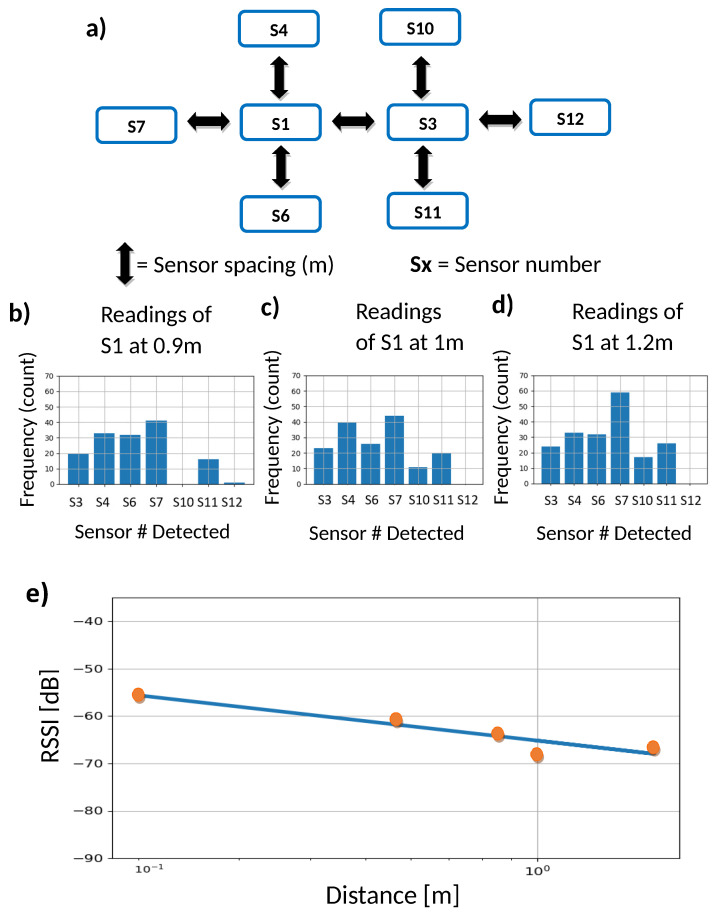
(**a**) Array of sensors in a pre-established star-like configuration. Initially, the distance between the nearest neighboring sensors is set at 0.2 m, then increased in 0.2 m intervals, until 1.2 m is reached. (**b**–**d**) Histograms of the number of measurements by S1 for three distance values. (**e**) Behavior of the average signal strength (RSSI) of all sensors versus distance in a semi-logarithmic plane. The solid line represents a linear regression that leads to the slope $a_{r}=-1.3$ ($R^{2}=0.90$).

To test the effectiveness of our device in a more realistic context, we have considered a non-simple configuration of a group of sensors, useful for statistical processing and elementary data verification. The configuration consists of eight sensors deployed in a star-like configuration (see Figure 9a). This configuration allows for observing the behavior of these sensors as the distance between sensors increases (as proposed below) and is a representation of sensor arrays in a real situation of social interaction. The distance between sensors of immediate neighbors is the same; initially, they are placed at 0 m between first neighbors, and then this distance increases by 0.20 m until it reaches 1.20 m (see Figure 9a), for a duration of 10 min per distance. This results in five different distance values.

The results of the number of measurements from three representative sensors for different values of distance are depicted in Figure 9b–d. Then, the distance between the neighboring sensors is increased while keeping the initial topology. As expected, when the distance between the sensors is small (D<0.20), most of the sensors detect each other (see Figure 9a), including those located at opposite ends of the array (S7 and S12). In contrast, for larger distances between first neighboring sensors (D>0.30) the closest neighboring sensors still register the highest power intensity, and some sensors no longer identify the presence of distant sensors.

For the case when the neighboring sensors are very close, most of the sensor pairs detect each other, as the distance increases, distant sensors are no longer detected and the number of measurements decreases significantly. To test the overall validity of the RSSI behavior in our sensor array, we considered all readings from all pairs of sensors for a given distance and calculate the mean values as a function of the distance. The results are shown in Figure 9c. As it can be seen in this semi-log plot, the intensity decays as the distance increases with a rate modulated by constants that can be determined with a linear regression (see figure caption for details). We also note that, as in experiment (i), there is a good agreement with the empirical Friis equation.

It is important to note that for this experiment, the information recorded by the GPS sensor and the accelerometer is complementary since the sensors were kept static and at a predetermined distance. In this sense, the information provided by the GPS and the accelerometer will be useful when considering dynamic situations.

### 4.4. Comparison with Other Sensors

The present sensor design allows the user to freely place the sensors at different distances. We have analyzed different configurations in which the range of distance between sensors is varied, which allowed us to determine sensing ranges that are useful for the identification of a proximity in a real context of social interaction. The data recorded by the sensors are consistent with the behavior reported for wireless sensor networks. Specifically, Table 2 presents a comparison of capabilities and elements between similar sensors reported in the literature and the current proposal. The proposed sensor with these features offers improved performance, range and connectivity, as well as acceptable power consumption. As shown in Table 2, several similar sensor developments were designed for use in different contexts but share similarities in design and operation.

## 5. Conclusions

In this paper we have presented a proximity sensing system, based on the integration of wireless devices, position sensors, and motion communication devices. The proposed embedded system is robust and can record sensor proximity in different configurations. Also, it can work in a stand-alone manner, remotely and with low cost. We have performed controlled tests with predetermined configurations, where we have been able to verify that neighboring sensors detect each other up to a certain distance threshold, relevant for normal social interactions between people. Additionally, we have verified that the intensity of the activity of the sensors, in terms of the distance between them, approximately follows the behavior predicted by well-accepted theoretical models. The system is functional and could potentially have a variety of uses. Although the proposed sensor represents an important step in collecting valuable information about social interactions in a school context, several aspects deserve further exploration. One possibility for future research could be to deploy the sensors in a wearable form (badge) during school hours on a group of undergraduate students in at least three stages of their studies. From this information, we propose to construct interaction networks, which would potentially allow a more complete understanding of how interaction patterns impact school performance, taking into account the dynamic nature of social interaction networks as the vehicle for the flow of information or knowledge among students. In addition, incorporating qualitative research methods, such as interviews and questionnaires, could provide a deeper understanding of relationships among students. By incorporating the nuances of their interactions and behaviors in a qualitative manner, it is possible to gain valuable insights into the underlying mechanisms that impact possible correlations between interaction patterns and school performance outcomes. In conclusion, our sensor represents a foundation for future research to contribute to a better understanding of the complex interactions between students and their possible relationship to school learning outcomes.

## Figures and Tables

**Table 1:** Stage characteristic table.

Parameter	Experiment (i)	Experiment (ii)
Country	Mexico	Mexico
City	CDMX	CDMX
Location	UPIITA-IPN	UPIITA-IPN
Start time (GMT-6)	17:04 h	22:20 h
End time (GMT-6)	20:04 h	20:20 h
Day	17 February 2024	21 March 2024
Wind Speed	50–59 km/h	40–60 km/h
Maximum Environmental Temperature	24.7 °C	28.9 °C
Minimum Environmental Temperature	10.5 °C	14.1 °C
Wifi name	WI-Fi IPN	Wi-Fi IPN
Internet Speed	53 Mbps	58 Mbps
Presence of humans	Yes	Yes

**Table 2:** List of sensors and their main characteristics reported in the literature. The first three rows correspond to sensors designed to identify social proximity or social interaction. The characteristics of the sensors include the space (indoor or outdoor) where the sensor was developed; the type of positioning system used by the sensor; as well as the type of internal measurement system for the systems; another parameter observed is the intensity indicator of the signal received. In this case, the question was whether it is used or not?. Another relevant data point is the number of sensors detected or the number of cards that maintain the communication. In addition to the range of coverage that the sensor has, we consider the following parameters: from 0 m to 1 m (short) or >1 m (high), and the cost.

Systems	Space	Positioning Systems	Inertial Measurement Unit	Received Signal Strength Indicator	Target BLE	Capacity Number Sensors	Range	Cost	Communication
[12]	I	IR	No	No	No	56	Short	High	Wifi
[17]	I	RFID	No	No	No	40	Short	High	Wifi
[18]	I	BLE	No	Yes	Yes	14	High	High	No
[51]	I	IPS	No	No	Yes	1	Short	Low	MQTT
[52]	E	No	No	No	Yes	1	High	Low	Wifi
[53]	I	UWB	No	No	Yes	1	High	High	Wifi
[54]	E	No	No	Yes	Yes	1	High	Low	MQTT
[55]	I	IPS	No	Yes	Yes	1	Short	Low	Wifi
[56]	I	UWB	Yes	No	No	1	Short	High	IoT
[57]	I	No	No	Yes	Yes	6	High	Low	Wifi
[58]	I and E	WMN and BLE	No	No	Yes	6	High	Low	IoT
**Proposal**	**E**	**GPS**	**Yes**	**Yes**	**Yes**	8	**Short**	**Low**	**Wifi**

## Data Availability

The data generated and analyzed in this study are included in this published article or are available from the corresponding author upon request.
